# Structure and dynamics of Type III periplasmic proteins *Vc*FhuD and *Vc*HutB reveal molecular basis of their distinctive ligand binding properties

**DOI:** 10.1038/srep42812

**Published:** 2017-02-20

**Authors:** Shubhangi Agarwal, Sanjay Dey, Biplab Ghosh, Maitree Biswas, Jhimli Dasgupta

**Affiliations:** 1 Department of Biotechnology, St. Xavier’s College, 30 Park Street, Kolkata 700016, India; 2 High Pressure & Synchrotron Radiation Physics Division, Bhabha Atomic Research Centre, Trombay, Mumbai 400085, India

## Abstract

Molecular mechanisms of xenosiderophore and heme acquisitions using periplasmic binding protein (PBP) dependent ATP-binding cassette transporters to scavenge the essential nutrient iron are elusive yet in *Vibrio cholerae*. Our current study delineates the structures, dynamics and ligand binding properties of two Type III PBPs of *V. cholerae, Vc*FhuD and *Vc*HutB. Through crystal structures and fluorescence quenching studies we demonstrate unique features of *Vc*FhuD to bind both hydroxamate and catecholate type xenosiderophores. Like *E. coli* FhuD, *Vc*FhuD binds ferrichrome and ferri-desferal using conserved Tryptophans and R102. However, unlike *Ec*FhuD, slightly basic ligand binding pocket of *Vc*FhuD could favour ferri-enterobactin binding with plausible participation of R203, along with R102, like it happens in catecholate binding PBPs. Structural studies coupled with spectrophotometric and native PAGE analysis indicated parallel binding of two heme molecules to *Vc*HutB in a pH dependent manner, while mutational analysis established the relative importance of Y65 and H164 in heme binding. MD simulation studies exhibited an unforeseen inter-lobe swinging motion in Type III PBPs, magnitude of which is inversely related to the packing of the linker helix with its neighboring helices. Small inter-lobe movement in *Vc*FhuD or dramatic twisting in *Vc*HutB is found to influence ligand binding.

Iron is an essential nutrient that pathogenic bacteria acquire to support the function of a number of enzymes crucial for survival, such as ribonucleotide reductase for the synthesis of DNA precursors, cytochromes for electron transport or tricarboxylic acid (TCA) cycle enzymes for energy production^1^. Since the concentration of free iron in tissues is extremely low (10^−18^ M)^2^, bacteria use various strategies to acquire Fe^3+^. Many infectious diseases, such as gonorrhea, malaria, tuberculosis and diarrheal infections depend on the expression of microbial acquisition mechanisms capable of competing with the host’s iron scavenging systems. The main strategies for iron acquisition include direct extraction of Fe^3+^ from specific iron-containing complexes synthesized by their hosts, such as lactoferrin, transferrin, haemoglobin or hemin^3^,^4^ and/or production of siderophores, small Fe^3+^-chelating molecules with high affinity and selectivity for Fe^3+^ ion^5^,^6^. The mechanisms of such ‘iron thievery’ of pathogenic bacteria have drawn special attention in recent years since these are being exploited for ‘Trojan Horse’ mechanism of antibiotic delivery to reduce permeability-mediated drug resistance^7^.

The iron uptake mechanism of *Vibrio cholerae*, the causative agent of diarrheal disease cholera, is currently insufficiently understood. Among the two major classes of siderophores, hydroxamates and catecholates, *V. cholerae* acquires iron through an indigenous catecholate siderophore, vibriobactin^8^. Some strains of non-epidemic *V. cholerae* responsible for wound infections and septicemia in susceptible people obtain enough iron from blood using iron acquisition systems other than vibriobactin^9^. Additionally, both vibriobactin synthesis and transport mutants cause disease in infant mice, further suggesting the presence of other mechanisms of iron acquisition within the host that include the transport and utilization of hemin and several xenosiderophores like ferrichrome, enterobactin and schizokinen^10^,^11^,^12^.

The uptake of iron compounds across the outer membrane of Gram-negative bacteria is facilitated by TonB-dependent receptors^1^. Active transport across the plasma membrane takes place through ATP binding cassette (ABC) transporters where cognate, periplasmic substrate binding proteins (PBPs) specifically bind a large variety of ligands^2^,^3^,^4^,^5^ and hydrolysis of ATP by the ATPase subunit provides energy for the transport of ligands to cytosol through the trans-membrane components^13^.

PBPs, in general, have bi-lobal structure and are categorized broadly into three structural classes^14^. While Types I and II PBPs have three and two inter-lobal β-strands or extended elements respectively, Type III PBPs are characterized by a single α-helical linker connecting the N- and C-terminal lobes^15^. Mostly, the siderophore and heme binding PBPs belong to Type III. Unlike Type I and II PBPs, which undergo large “Venus flytrap” conformational changes upon ligand binding, Type III PBPs are known as relatively more rigid molecules, although in some proteins like FepB of *E. coli* or HtsA of Gram-positive bacteria *Staphylococcus aureus*, ligand binding is accompanied by significant loop movements^16^,^17^. FhuD of *Escherichia coli (Ec*FhuD), another Type III PBP, binds hydroxamate siderophores such as ferrichrome, desferal, fungal siderophore coprogen and the structurally related antibiotic albomycin^2^,^18^. Interestingly, despite a high degree of structural conservation, PBPs such as *Ec*FhuD, YfiY of *Bacillus cereus* or FhuD2 of *Staphylococcus aureus* have evolved with critical siderophore binding residues on either the C-lobe, N-lobe or both^19^.

Available structural evidences on heme binding PBPs, on the other hand, suggest variable mode of heme binding in different bacteria. While ShuT of *Shigella dysenteriae (Sd*ShuT) and PhuT of *Pseudomonas aeruginosa (Pa*PhuT) share common architecture and bind one heme molecule in a narrow cleft between the N- and C-terminal lobes, HmuT of *Yersinia pestis (Yp*HmuT), having similar overall structure, contains a significantly wider central cleft capable of accommodating two stacked heme molecules^20^,^21^.

In *V. cholerae*, the molecular details of siderophore binding by the PBPs are restricted to the structures of catecholate specific ViuP^22^ and VctP^23^ while mechanisms of hydroxamate siderophores or heme binding are unknown yet. Here we report the structures, dynamics and ligand binding properties of (i) a putative ferrichrome binding PBP, *Vc*FhuD (*Accession code: A0A0H6E7H0*) that belongs to the ABC transporter genes *fhuBCD*^24^ and (ii) the heme binding PBP, *Vc*HutB (*Accession code: A5F0S5*) belonging to *hutBCD* of *V. cholerae* genome^25^. The crystal structures of *Vc*FhuD and *Vc*HutB, together with Molecular Dynamics (MD) simulations and biochemical analysis, provide first quantitative overview of xenosiderophores and heme binding by the PBPs in *V. cholerae. Vc*FhuD has shown a unique property of binding both hydroxamate and catecholate siderophores, mechanistic implication of which has been addressed here. Structure of apo-*Vc*HutB coupled with heme-protein interaction studies through native PAGE and spectrophotometry on the wild type and mutant proteins have illuminated distinctive features of heme binding by identifying the differences in binding mechanism of two heme molecules by *Vc*HutB and *Yp*HmuT. Additionally, MD simulation results have identified distinguishing inter-lobe movements of Type III PBPs that lead to efficient and diverse ligand binding.

## Results

### Growth revival of *V. cholerae O395* by exogenous siderophores and heme

**Figure 1:**
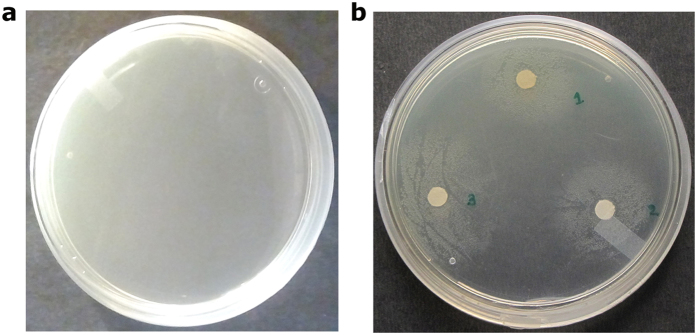
(**a**) Effect of EDTA on growth of *V. cholerae* O395 strain; (**b**) Distinct zones of growth observed around filter paper disks soaked with (1) heme, (2) ferrichrome and (3) ferri-desferal in the presence of EDTA.

We have investigated the capability of *V. cholerae O395* strain to utilize Fe^3+^ bound to the siderophores and heme using growth assay. For this purpose, we have used EDTA (ethylene diamine tertaacetic acid), the versatile chelator that binds both Fe^3+^ and Fe^2+^ ions. 150 μg of EDTA per ml at 1 × 10^4^ bacteria per ml caused complete inhibition of growth of *V. cholerae* (Fig. 1a). Precise zones of growth were, however, observed around the disks soaked with heme, ferrichrome and Fe^3+^ treated deferoxamine mesylate (ferri-desferal) in a metal ion depleted plate which establish that these iron compounds can efficiently be used by *V. cholerae O395* strain to restore growth (Fig. 1b).

### Overall structure of *Vc*FhuD in apo and ferri-Desferal bound states

**Figure 2:**
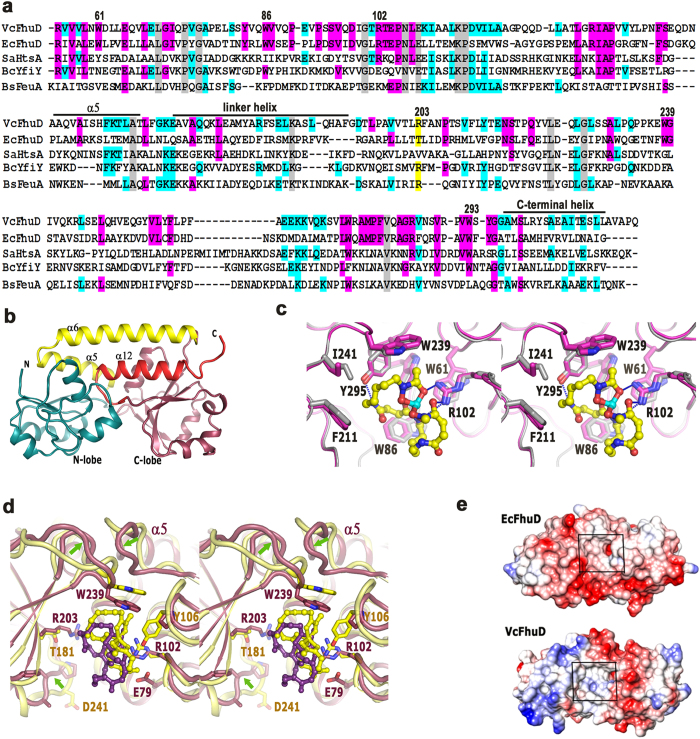
(**a**) Sequence alignment of *Vc*FhuD from *V. cholerae, Ec*FhuD from *E. coli, Sa*Htsa from *S. aureus, Bc*Yfiy from *B*. cereus and *Bs*FeuA from *B. subtilis*. Numbering is based on *Vc*FhuD sequence. Important motifs/residues are indicated by *black bars* and/or marked. Conserved residues are shown in gray. (**b**) Structure of apo-*Vc*FhuD. (**c**) Stereo view of the superposition of holo and apo *Vc*FhuD shown in magenta and white. The hydrophobic and polar interactions of *Vc*FhuD with ferri-desferal are also shown here. (**d**) Stereo view of the comparison of ferri-desferal binding to *Vc*FhuD (violet) and *Ec*FhuD (yellow). Significant differences in loop conformation are shown by arrows. (**e**) **C**oulombic potential of *Ec*FhuD and *Vc*FhuD surfaces.

*Vc*FhuD shares 30% sequence identity with its nearest structural homolog *Ec*FhuD where the hydroxamate siderophore binding residues are found to be conserved (Fig. 2a). Tris-catecholate siderophore binding protein FeuA from *Bacillus subtilis*^26^, YfiY from *Bacillus* cereus or *Staphylococcus aureus Lipoprotein* HtsA^17^ show about 27% sequence identity with *Vc*FhuD. However, overall sequence identity among these PBPs is as low as 3.6% (Fig. 2a). Interestingly, vibriobactin binding PBP ViuP of *V. cholerae*^22^ exhibits only 15% identity with *Vc*FhuD.

We have solved the crystal structure of *Vc*FhuD in apo and ferri-desferal bound states. Two polypeptide chains of the asymmetric unit of apo-*Vc*FhuD are almost identical with an rmsd of 0.3 Å. *Vc*FhuD has a typical bi-lobal structure where the two lobes are connected by a 25-residue linker helix designated as α6 (Fig. 2b). The N-terminal lobe is comprised of a twisted five-stranded parallel β-sheet connected by the α-helices whereas the C-terminal lobe has a mixed β-sheet surrounded by the α-helices (Fig. 2b). Helix at the end of N-terminal lobe (α5) and the extreme C-terminal helix (α12) provide stability to the linker helix through hydrophobic and polar interactions. The siderophore binding site is located in the shallow cleft between the N- and C-terminal lobes (Fig. 2b).

Superposition of holo-*Vc*FhuD on the apo structure produces an rmsd of 0.4 Å and no significant change, either in the overall structure or at the ligand binding pocket, is observed between the two. The conformation of the ligand binding residues almost remains unaltered upon ferri-desferal binding, as observed in Fig. 2c. All four molecules of holo-*Vc*FhuD of the asymmetric unit show unambiguous electron density of the ligand, ferri-desferal (Fig. S1). Similar to ferri-desferal bound *Ec*FhuD structure, the mesylate (OSO_2_CH_3_) portion of the siderophore could not be located in the electron density probably because of its disordered nature.

### Properties of ligand binding site and ferri-desferal binding

The ligand binding cleft of *Vc*FhuD is primarily hydrophobic and made of W61, W86 of the N-lobe helices, W239 and W293 from the loop regions of the C-lobe and F211 and I241 of the C-lobe helices (Fig. 2a,c). R102 remains pre-oriented for the ligand binding by hydrophobic packing with W61 (Fig. 2c) and the salt bridge interactions with E79 (Fig. 2d). Corresponding residue, R84 of *Ec*FhuD, is found to be salt bridged with E42. The ferrioxamine portion of ferri-desferal snugly fits in the binding cleft of *Vc*FhuD (Fig. 2c,d). Two hydroxamate carbonyl oxygens of ferri-desferal are hydrogen bonded by R102 (Fig. 2c). The other hydroxamate carbonyl oxygen solely serves the purpose of coordinating Fe^3+^ of ferri-desferal. Y295 forms hydrogen bond with the backbone of ferri-desferal (Fig. 2c).

Ferri-desferal bound *Vc*FhuD superposes on its *E. coli* counterpart (PDB code: 1K2V) with an rmsd of 1.6 Å. Although ferri-desferal binding residues are primarily conserved in these two PBPs, the loops surrounding the binding site differ significantly (Fig. 2d). In *Vc*FhuD, the loop that connects α5 with the N-lobe is more inclined toward the ligand binding pocket by ~8 Å (Fig. 2d). As a result, the loop of the C-lobe that possesses W239 experiences a lateral shift towards the pocket and the ferri-desferal bound to *Vc*FhuD is shifted outward compared to that bound to *Ec*FhuD (Fig. 2d). However, because of similar lateral shift of the crucial residues, which are responsible for polar interactions and hydrophobic packing with ferri-desferal, the binding of this ligand to *Vc*FhuD is not compromised (Fig. 2d). The contribution made by Y106 of *Ec*FhuD in ferri-desferal binding is not expected in *Vc*FhuD since P123 occupies the corresponding position of the later (Fig. 2d).

Important variations in surface electrostatics around the binding pocket are observed between *Ec*FhuD and *Vc*FhuD (Fig. 2e). The charge of the residues surrounding the binding site of *Ec*FhuD is predominantly negative, making it unsuitable for binding the negatively charged catecholate siderophores^2^,^18^. Some of such negatively charged residues of *Ec*FhuD namely, E42, E213, T181, D239, D241 are replaced by non-polar, zwitterionic and basic residues in *Vc*FhuD (Fig. 2a). This, together with the adjacent shorter and non-polar loop makes the binding site of *Vc*FhuD apolar with slightly basic nature and accessible both for the catecholate and hydroxamates siderophores (Fig. 2d,e).

### Ligand binding affinity of *Vc*FhuD

**Figure 3:**
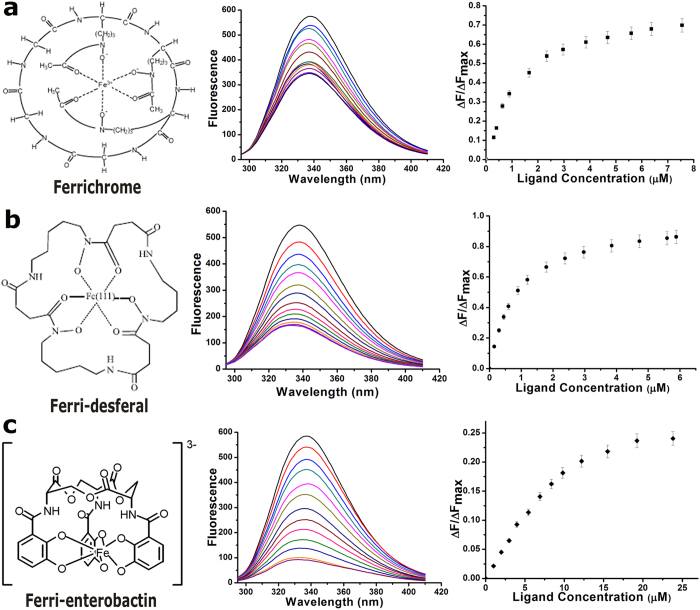
Structures of (**a**) ferrichrome^48^, (**b**) ferri-desferal and (**c**) ferri-enterobactin^49^ are shown in the left panel. *At middle* are the fluorescence quenching graphs of *Vc*FhuD in the presence of aforesaid ligands. *At right* are the plots of Δ*F*/Δ*F*max *versus* ligand concentration (in μM).

We have calculated the binding affinity of *Vc*FhuD to (i) Fe^3+^ treated ferrichrome, (ii) Fe^3+^ treated deferoxamine mesylate and (iii) Fe^3+^ treated enterobactin by measuring the intrinsic fluorescence quenching (λ_exc_ = 280 nm, λ_em_ = 295–410 nm), accounting the contributions of tryptophans and tyrosines present at the ligand binding site. Ferrichrome and ferri-desferal have shown significant binding with *Vc*FhuD with the dissociation constant (K_d_) values of 1 ± 0.226 μM and 0.6 ± 0.015 μM respectively (Fig. 3a,b). Interestingly, considerable interaction is also observed between catecholate siderophore ferri-enterobactin and *Vc*FhuD. Since binding of ferri-enterobactin to a hydroxamate siderophore specific PBP is an outstanding observation, we have verified this binding through near-UV CD. The gradual increase of the positive band at 268 nm with increasing amount of the ferri-enterobactin, keeping protein amount constant, indicates interactions between the two (Fig. S2). However, for quantitative measurements of affinity, fluorescence quenching data were used which produce a K_d_ of 5.5 ± 0.414 μM (Fig. 3c). Our results indicate that *Vc*FhuD has the unique property of binding both hydroxamate and catecholate siderophores.

### Structural uniqueness of *Vc*HutB

**Figure 4:**
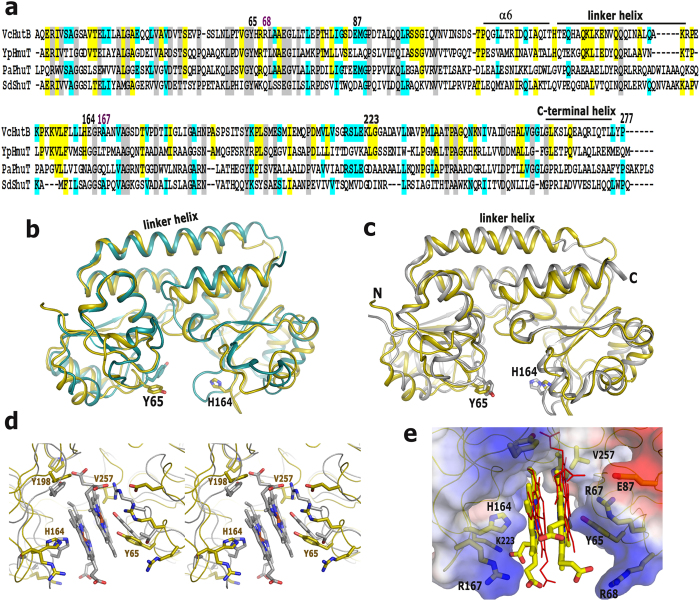
(**a**) Sequence alignment of *Vc*HutB with *Yp*HmuT from *Yersinia pestis, Pa*PhuT from *Pseudomonas aeruginosa* and *Sd*ShuT from *Shigella dysenteriae*. Numbering is of *Vc*FhuD sequence. Important motifs are indicated by *black bars* and marked. Conserved residues are marked in gray. (**b**,**c**) Overall structure of apo-*Vc*HutB is compared with (**b**) apo-*Sd*ShuT and (**c**) apo-*Yp*HmuT. (**d**) Stereo view of the superposition of *Yp*HmuT on apo-*Vc*HutB showing that heme binding in this manner may cause clashes with *Vc*HutB loops. (**e**) Docking of two heme molecules (shown as yellow sticks) in the binding site of *Vc*HutB. Conformation of the heme molecules bound to *Yp*HmuT is shown by red lines.

Heme uptake from the host is another important aspect that contributes significantly to iron acquisition mechanisms of bacteria. *Vc*HutB has been identified as the exclusive heme binding PBP of *V. cholerae* that shares 32%, 34% and 37% sequence identity with the other structurally characterized heme binding PBPs, *Sd*ShuT, *Pa*PhuT and *Yp*HmuT respectively (Fig. 4a).

The structure of apo-*Vc*HutB has been determined upto 2.4 Å resolution. Although *Vc*HutB has shown highest identity with *Yp*HmuT (Fig. 4a), only apo-*Sd*ShuT (PDB code: 2RG7)^20^ produced an acceptable MR solution for *Vc*HutB. Apo-*Vc*HutB superposes on apo-*Sd*ShuT and apo-*Yp*HmuT with an rmsd of 1.9 Å and 2.1 Å respectively showing significant differences in the loop regions around the ligand binding site (Fig. 4b,c). *Sd*ShuT binds one heme molecule in a narrow cleft using Y67^20^. The wider cleft of *Yp*HmuT, on the other hand, accommodates two heme molecules stacked in an anti-parallel fashion where Y70 and H167 anchor Fe^3+^ of two separate heme molecules^21^. *Vc*HutB also contains two potential heme binding residues Y65 and H164, located likewise on either side of the cleft with suitable micro-environment to bind heme (Fig. 4a,c). Additionally, the adjacent R67 is in a position to stabilize the tyrosinate formed by Y65. The heme binding pocket of *Vc*HutB is even wider than *Yp*HmuT with an average Cα distance of 20 Å between Y65 and H164 which is 15 Å for *Yp*HmuT.

Structural comparison of YpHmuT and *Vc*HutB suggests that although the ligand binding pocket of *Vc*HutB is suitable to accommodate two heme molecules, the mode of heme binding may differ from *Yp*HmuT. Two loops of *Vc*HutB which possess Y198 and V257 respectively are oriented differently to cause steric clashes if the heme molecules would bind in a manner similar to *Yp*HmuT (Fig. 4d). Being already tightly packed, these loops of *Vc*HutB are apparently incapable of changing conformation to accommodate heme. Our knowledge based docking of two heme molecules in the binding pocket of *Vc*HutB proposes parallel mode of heme binding with probable participation of the unique basic residues R68, R167 and K223 which are poised to stabilize the propionate groups (Fig. 4a,e).

### Shift of Soret band of heme upon *Vc*HutB binding

**Figure 5:**
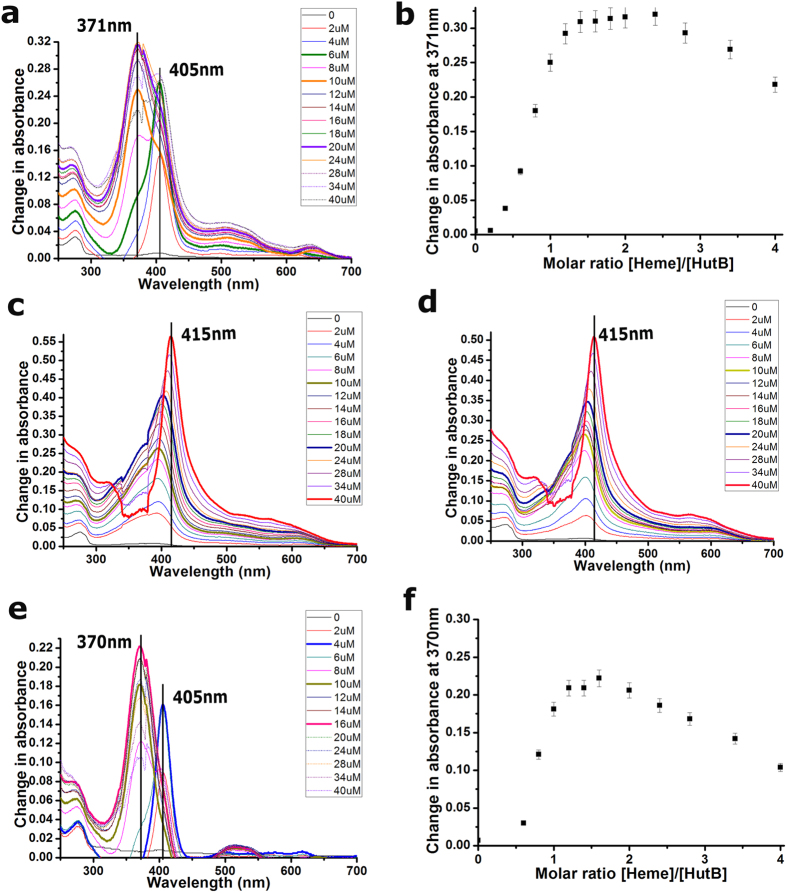
Hemin binding by *Vc*HutB is observed by UV/VIS spectroscopy. (**a**) *Vc*HutB at pH 8.0 (no protein in the reference cuvette) was titrated with increasing amounts of hemin. Difference spectra (reference subtracted from sample) are shown for selected heme concentrations (colours as indicated). (**b**) Saturable heme binding is shown by plotting change in absorbance at 371 nm versus hemin concentration measured at pH 8.0. (**c**,**d**) Difference spectra for *Vc*HutB-Y65A and *Vc*HutB-Y65F at pH 8.0. (**e**) Titration of *Vc*HutB at pH 7.0 with increasing amounts of hemin, by same method as of (**a**). (**f**) Change in absorbance at 370 nm versus hemin concentration indicates saturable heme binding at pH 7.0.

We attempted to estimate the relative affinity of heme for *Vc*HutB spectrophotometrically by performing hemin titration experiments. The absorption spectrum of hemin features a peak in the Soret band region around 385 nm. Hemin was incrementally titrated to free *Vc*HutB at pH 8.0 and the spectral range between 250–700 nm was measured. *Vc*HutB alone does not show absorption in Soret region but when it was mixed with hemin, the Soret band initially shifted towards right with maxima at 405 nm, followed by a blue-shifted peak at 371 nm, suggesting *Vc*HutB mediated perturbation of the electronic structure of the hemin iron which is indicative of interactions between the two (Fig. 5a). The protein concentration for this experiment was 10 μM. The highest intensity at 405 nm was observed with hemin concentration of 6 μM and the 2^nd^ peak at 371 nm was found to be saturated with 20 μM of hemin (Fig. 5a). The distinct absorption maxima, one at 405 nm and another at 371 nm, point towards two binding events with participation of H164 and Y65, as observed previously in case of *Yp*HmuT^21^. Change in absorbance at 371 nm versus hemin concentration indicates saturable heme binding to VcHutB (Fig. 5b). Changes in the slope of this graph shows a plateau at the top with two apparent maxima at VcHutB:hemin molar ratios of ~1:1.5 and ~1:2.5 which probably points towards two binding events (Fig. 5b).

### Effect of mutations and pH on the shift of hemin Soret band

We performed the same experiment with the mutants *Vc*HutB-Y65A and Y65F. Almost no peak shift occurred upto 20 μM of hemin and then gradual red shift took place upto 415 nm with an increasing concentration of hemin upto 40 μM (Fig. 5c,d). No blue shifted peak was observed for either of these mutants. The results indicate that although H164 is capable of recognizing heme, interactions are not efficient enough in the absence of Y65. Furthermore, to investigate the effect of pH on heme binding, same experiment was carried out with wild type *Vc*HutB at pH 7.0 (Fig. 5e,f). We have obtained similar Soret shift as observed in case of pH 8.0. However, in contrast to pH 8.0 (Fig. 5a,b), the highest intensity at 405 nm was observed here with hemin concentration of 4μM and a saturation of the 2^nd^ peak at 16 μM. The molar ratio graphs indicate saturable heme binding to *Vc*HutB, where the maxima at pH 7.0 are observed at protein:heme ratio of ~1:1.3 and ~1:1.6 (Fig. 5f). Presumably, incomplete deprotonation of H164 causes weaker heme binding at pH 7.0.

### Native PAGE shows binding of two heme molecules to *Vc*HutB

**Figure 6:**
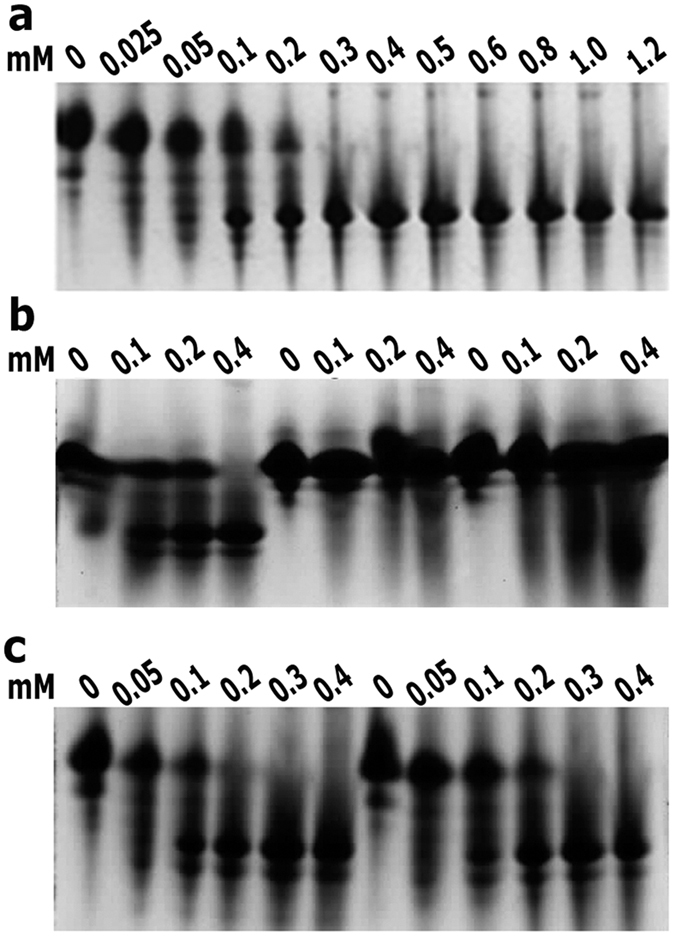
Native-PAGE analysis. (**a**) 0.2 mM *Vc*HutB titrated with increasing concentration of hemin. (**b**) Comparison of the titration of 0.2 mM of *Vc*HutB, *Vc*HutB-Y65A and *Vc*HutB-Y65F. (**c**) Comparison of the titration of 0.2 mM *Vc*HutB at pH 7.0 (first 6 lanes) and at pH 8.0 titrated with same hemin concentrations as done with pH 8.0 (last 6 lanes). In each case, hemin concentration is mentioned in mM at the top of the panel. Maximum region of the gel is shown in each panel, excluding stacking gel and dye font.

To obtain an affinity estimate, we studied the interactions between *Vc*HutB and hemin by native PAGE, as it was employed previously^27^. Hemin-loaded *Vc*HutB, as judged by visual inspection of Coomassie blue-stained gels, migrates faster in native PAGE than the apo form, reflecting significant compactness of *Vc*HutB upon heme binding, as observed in case of the heme binding PBP, HbpA of *Haemophilus influenza*^27^. Experiments were carried out at pH 8.0 with 0.2 mM of *Vc*HutB and a complete shift from apo to holo band occurs with 0.4 mM of hemin further indicating 1:2 binding between *Vc*HutB and heme (Fig. 6a). It was known that as the hemin concentration approaches its K_d_ value, the protein band splits up, with about half of it migrating faster because of complexation with hemin^28^. Based on densitometric scanning of Coomassie blue-stained gels we have determined the relative amounts of apo-*Vc*HutB as a function of increasing hemin concentrations. By plotting the optical densities of the bands on gel and fitting the data in non-linear curve of GraphPad Prism 7.01, the apparent dissociation constant (K_d_) of 200 μM has been calculated for the hemin-*Vc*HutB binding. The K_d_ value indicates that similar to ShuT/PhuT or HbpA, heme is not very tightly held by *Vc*HutB. We repeated the same experiments with *Vc*HutB mutants Y65A and Y65F and the results suggest that the compactness acquired upon heme binding is the lowest for Y65A and little higher for Y65F although it is still much lesser than the wild type (Fig. 6b). Native PAGE at pH 7.0 showed a protein:heme stoichiometry that corroborates the observations of spectroscopic studies (Fig. 6c).

### MD simulation and principal component analysis (PCA) reveal unique inter-domain movement in *Vc*FhuD and *Vc*HutB

**Figure 7:**
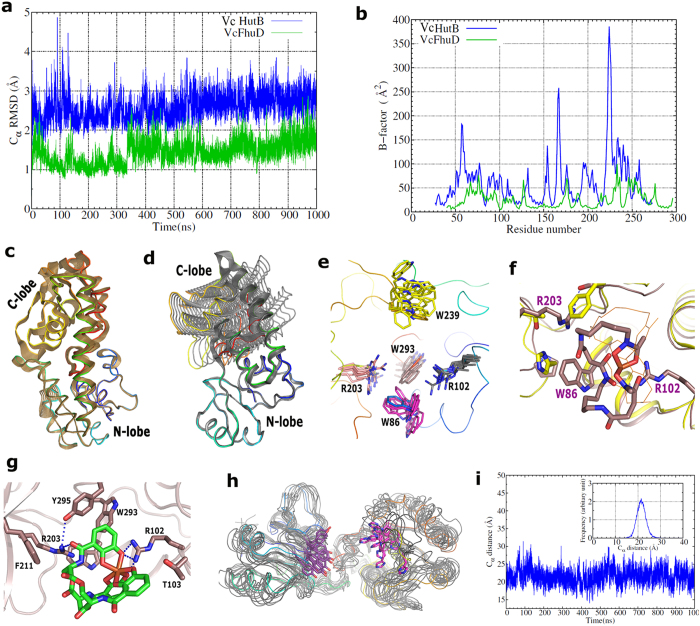
(**a**) Root Mean Square Deviation (RMSD) for the C-alpha atoms is shown for both *Vc*HutB (blue) and *Vc*FhuD (green) for the full simulation range. (**b**) The Root Mean Square Fluctuation (RMSF) of the C_α_ atoms was calculated from the simulation trajectories and then converted into the B-factor of the C_α_ atoms. *Vc*HutB protein is clearly showing more fluctuations than *Vc*FhuD. Visualization of (**c**) VcFhuD and (**d**) VcHutB, as predicted by PCA. The conformations are aligned with respect to the N-lobe to show the inter-domain movement. (**e**) Snapshots of MD trajectories of *Vc*FhuD are superimposed to show the dynamics of the residues at the binding site. (**f**) Superposition of ferri-desferal bound *Vc*FhuD structure on that of *Ec*FhuD to show the spatial disposition of different important residues at the binding site. (**g**) Docking of ferri-enterobactin at the ligand binding pocket of *Vc*FhuD. (**h**) Superposition of the snapshots of MD simulation trajectories to show the movement of Y65 and H164 in *Vc*HutB. (**i**) MD simulations show the dynamics of the substrate binding region between the N and C-lobes of *Vc*HutB. The distance between the C_α_ atoms of Y65 and H164 is shown against the simulation time (inset).

To understand the dynamic nature of *Vc*FhuD and *Vc*HutB, we carried out MD simulations on the apo structure of both the PBPs for 1000 ns. Both *Vc*FhuD and *Vc*HutB are found to be mechanically stable as seen from their RMSD values over the simulation time (Fig. 7a). B-factors, averaged over 1000 ns of MD simulation trajectories demonstrates that while *Vc*FhuD is moderately stable, some of the segments of *Vc*HutB, such as majority of the C-lobe including H164, the loop that connects linker helix with the C-lobe and the loop region adjacent to Y65 show significant dynamics (Fig. 7b). To bring out the collective internal motions in the proteins under consideration, we have carried out Principal Component Analysis (PCA)^29^ on the MD trajectories for up-to 500 ns. For PCA, all the atoms in the protein were considered except the five residues from the terminals due to their excessive motion. The covariance matrix was calculated using the option “covar” and the eigenvectors were analyzed using ‘anaeig’ in Gromacs. Before the covariance matrix was built, the overall translational and rotational motion was eliminated as they are irrelevant for the analysis of the internal motions. The eigenvalues of the covariance matrix were calculated and arranged in order of decreasing value. The motion along the first five eigenvector directions is shown by projecting the trajectory onto these individual eigenvectors against time (Fig. S3). Since the eigenvalues are the average square displacements, the first eigenvectors represent the largest positional deviation, which is pictorially presented in Fig. 7c,d. To date, ‘open’ and ‘closed’ conformations related to the substrate binding mechanism was observed in Type III siderophore binding PBPs like HtsA of *S. Aureus* or FepB of *E. coli*^16^,^17^. In contrast to that, our results of MD trajectories and resultant PCA revealed a unique twisting movement between the N- and C-lobes, extent of which varies dramatically from one Type III PBP to the other.

## Discussion

Blocking and interfering with the iron acquisition systems could disrupt bacterial iron homeostasis and thus suppress bacterial growth. Our observations of growth assay (Fig. 1) established that heme, ferrichrome and ferri-desferal act as efficient iron sources and are utilised by *V. cholerae O395* to restore growth. This observation encouraged us to investigate the mechanism of uptake of these iron compounds by *Vc*FhuD and *Vc*HutB inside *V. cholerae*.

*Vc*FhuD efficiently binds hydroxamate siderophores ferrichrome and ferri-desferal (Fig. 3a,b). Crystal structure showed that *Vc*FhuD possesses a preformed pocket for accommodating hydroxamate siderophores. Binding of ferri-desferal in this pocket is incurred by hydrophobic packing using four conserved tryptophans, W61, W86, W239 and W293, supported by the polar interactions with R102 and Y295 (Fig. 2c). Gallichrome and albomycin binding to *Ec*FhuD using R84 (which corresponds to R102 of *Vc*FhuD) and four conserved tryptophans^2^,^18^ suggest that these ligands are expected to fit in the binding pocket of *Vc*FhuD as well. MD simulations and the resultant PCA indicated that although the overall inter-lobe movement is negligible in case of *Vc*FhuD (Fig. 7c), variable local and subtle movements of different crucial amino acids occur here (Fig. 7e). R102 executes minimum fluctuations with retention of the salt bridge with E79 almost in all the frames. On the other hand, variable fluctuations are executed by the tryptophans: while movement is negligible for W61 and W293, relatively higher fluctuations are exhibited by W86 and W239 (Fig. 7e). Presumably, variable ligand binding by *Vc*FhuD is facilitated by the movement of key residues by creating enough room to accommodate different hydroxamate siderophores.

So far, most of the reported PBPs only accept single type (either hydroxamate or catecholate) siderophore. Contrary to that, along with binding the cognate hydroxamate siderophores, *Vc*FhuD also binds catecholate xenosiderophore ferri-enterobactin with substantial affinity (K_d_ of 5.5 ± 0.414 μM) (Fig. 3c). The intriguing question raised is if the hydroxamate siderophore binding residues are predominantly conserved in *Ec*FhuD and *Vc*FhuD then what is making the later capable of binding ferri-enterobactin ? Interestingly, unlike *Ec*FhuD, the binding pocket of *Vc*FhuD is primarily made of non-polar and positively charged residues that exert no repulsion to the catecholate siderophores (Fig. 2e). Ferri-enterobactin bound structure of *E. coli* FepB shows that along with some hydrophobic residues, three diagonally opposite Arginines critically interact with ferri-enterobactin^16^. *Vc*FhuD possesses a unique R203 located opposite to R102 (Fig. 7f). Notably, R203 of *Vc*FhuD corresponds to T181 of *Ec*FhuD which is known to add negative charge to the binding site of *Ec*FhuD^18^. On the other hand, R203 corresponds to R180 of *Bs*FeuA (Fig. 2a) which is seen to play a predominant role in binding ferri-enterobactin (PDB code: 2XUZ)^26^. MD simulation trajectories showed that although R203 forms hydrophobic lining for ferri-desferal in the ligand binding pocket of *Vc*FhuD and its terminal basic group hydrogen bonds with the side chain hydroxyl group of S294, R203 has enough potential to orient towards the ligand binding pocket during dynamics. In fact, a concerted movement of W86 creates space for such re-orientation of R203 (Fig. 7e). Docking of ferri-enterobactin in the ligand binding pocket revealed that the non-negative nature of the binding site, together with the contributions of R102 and R203, render *Vc*FhuD suitable for ferri-enterobactin binding (Fig. 7g).

With the rapid rise in bacterial resistance to antibiotics, the cooperative behaviour in microbial communities needs to be urgently understood for the development of novel drugs to control infections caused by resistant bacteria and utilization of ferri-enterobactin by *V. cholerae* as iron source is an important phenomenon in this respect. No PBP was identified in *V. cholerae* so far which is capable of efficiently binding ferri-enterobactin. Therefore, the broader specificities of *Vc*FhuD towards hydroxamate siderophores like ferrichrome and ferri-desferal and the catecholate siderophores ferri-enterobactin, as revealed in our study, may provide *V. cholerae* with competitive advantage for survival. In turn, because of this property, *Vc*FhuD may emerge as a potential drug target.

Additionally, our observations help to better understand the mechanism of variable heme binding by the PBPs in different bacterial species. Binding of two heme molecules with efficient participation of both Y65 and H164 provides compactness to the structure of *Vc*HutB leading to faster migration in native PAGE (Fig. 6). Moreover, heme binding to *Vc*HutB is found to be pH dependent and heme affinity reduces with the decrease in pH from 8.0 to 7.0 (Figs 5 and 6). Such pH dependence may be attributed to the incomplete deprotonation of H164 at low pH causing inefficient heme binding. Appearance of major Soret peak at 405 nm in low heme concentration, which shifts to 371 nm at high heme concentrations, together with the results of native PAGE at different pH, suggest that even though H164 is capable of recognising heme, efficient binding must require Y65. Although binding of two heme molecules in anti-parallel fashion was structurally established in *Yp*HmuT, our docking study revealed that the micro-environment of the heme binding site indulge in binding of two hemes in parallel fashion to *Vc*HutB with additional participation of the unique basic residues R68, R167 and K223 to stabilize the propionate groups (Fig. 4d).

The physiological significance of binding of two heme molecules *in-vitro* to *Vc*HutB (or to *Yp*HmuT) remains to be established. The size of the periplasmic entrance to the translocation pathway of HmuUV of *Yersinia pestis*, whose sequence is highly similar to *Vc*HutCD, is narrower than BtuCD of *E. coli*^30^ which in turn point towards plausible translocation of one heme molecule by *Vc*HutCD during single reaction cycle. The binding of two heme molecules by *Vc*HutB, may thus be attributed to storage purpose which occurs in a pH and concentration dependent manner. Notably, heme storage is observed in heme degrading protein MhuD of *Mycobacterium tuberculosis* in non-iron depleted situation^31^.

Most siderophore and heme transporters are Type III PBPs. Although most of the Type III PBPs have a relatively stable bi-lobal arrangement with a linker helix, the sequence diversity within the superfamily is so high that most of the PBPs acquire significantly different non-superimposable structural folds, as observed in case of *Vc*FhuD and *Vc*HutB, the apo structures of which superpose with an rmsd of 15 Å. In contrast to the ‘open’ to ‘close’ lobe movement observed previously for Type III PBPs upon ligand binding, our MD simulation results and the resultant PCA revealed an inter-lobe twisting motion, magnitude of which vary dramatically. *Vc*HutB undergoes dramatic inter-lobe swinging which is more vigorous for the C-lobe. While an ‘in-out’ movement is observed for Y65, movement of H164 seems to be more chaotic (Fig. 7h). During MD simulations, the distance between the Cα atoms of Y65 and H164 varies between 15 to 26 Å with a maximum at 22 Å (Fig. 7i). Variation in proximity of these two crucial residues might be the determinant factor for efficient heme binding. Critical analysis of the simulation trajectories suggests that dynamics of these two Type III PBPs are primarily controlled by the interactions of the linker helix with its two neighbouring helices. Interestingly, the linker helix, along with the two adjacent helices shows maximum sequence variations (Figs 2a and 4a). The interactions among these helices are quite extensive and robust in case of *Vc*FhuD because of which *Vc*FhuD has emerged as a relatively rigid molecule with negligible inter-lobe movement (Fig. 7c). On contrary, the linker helix of *Vc*HutB is one turn shorter than *Vc*FhuD with a highly flexible loop at the C-terminal part of the linker helix (Fig. 7b). Furthermore, in *Vc*HutB, the neighbouring helices are not properly parallel to the linker helix, leading to less extensive packing among them. As a result, *Vc*HutB is more dynamic with drastic inter-lobe twisting and variable proximity between Y65 and H164, which is expected to be an important factor in regulation of heme binding and/or exchanging. Collectively, our current study produces first mechanistic view of xenosiderophores and heme binding by the PBPs in *V. cholerae* including the relationship between the dynamic behaviour of Type III PBPs and ligand binding properties. Further investigations in this direction with more Type III PBPs would also be of interest to enrich the ‘Trojan Horse’ mechanism of drug delivery.

## Methods

### Growth assay

Growth assays were performed to test the ability of *V. cholerae* to utilize various compounds as iron sources. Mid-log-phase LB broth culture was plated onto LB agar - streptomycin plate containing 150 μg of EDTA per ml. Filter paper disks (3 mm in diameter) containing 460 μM of hemin chloride, 200 μM of ferri-desferal and 250 μM of ferrichrome were placed on the plates and revival of growth around the disks was examined after 16 h of incubation at 37 °C.

### Cloning, overexpression and purification

Gene sequence of *Vc*FhuD without the signal peptide(N-terminal 54 amino acids) was cloned into kanamycin resistant pET28a^+^ (Novagen) vector using specific primers (forward primer: 5′-GGAATTCGCATATGCGAGTGGTGGTGCTGAACTGGGATC-3′, and reverse primer: 5′-CATTCGGGATCCTCATGATTGCGGAGCCACCGCCAGC-3′). The primers were synthesized (NeuProCell) with *NdeI* and *BamHI* restriction enzyme sites. Chromosomal DNA of *V. cholerae* strain O395, isolated using the protocol described in the Molecular Biology Laboratory Manual of UMBC (http://userpages.umbc.edu/~jwolf//methods.html), was used as the template to amplify the region encoding *fhuD*. The 795 bp *fhuD* PCR amplicon and the pET28a^+^ vector with *NdeI* and *BamHI* restriction sites were ligated using T4 DNA ligase and the appropriate clones were selected using *E. coli* XL1-Blue cells with kanamycin resistance. The construct was verified by restriction digestion analysis and commercial DNA sequencing.

Protocol of cloning of *Vc*HutB (residues 24–277 excluding signal peptide; 254 aa) in *E. coli* XL1-Blue cells in presence of the antibiotic, kanamycin, as a fusion protein having 6 × His-tag at the N-terminus has been described in Agarwal *et al*.^25^. Matured *Vc*FhuD (residues 55–319 excluding signal peptide; 265 aa) and *Vc*HutB were overexpressed in *E. coli* BL21 (DE3) cells in the presence of antibiotic, kanamycin, by Isopropyl β-D-1-thiogalactopyranoside (IPTG) induction and then purified to homogeneity by Ni-NTA affinity chromatography as per protocol reported in Agarwal *et al*.^25^. Mutants of *Vc*HutB, Y65A and Y65F were prepared by two-step PCR. The mutants were verified by commercial sequencing and purifications were done following the same protocol as wild type *Vc*HutB.

### Crystallization and diffraction data collection

Crystals of the PBPs were grown at 293 K using the hanging drop vapour diffusion method. Apo-*Vc*FhuD crystals were obtained when 2 μl of the protein (45 mg/ml) and 2 μl of precipitant solution consisting of 18% (w/v) PEG 6000, 0.1 M MES (pH 6.0) were equilibrated against a reservoir solution of 34% (w/v) PEG 6000, 0.1 M NaCl and 0.1 M Tris (pH 7.0) for one month. Holo-*Vc*FhuD was prepared in a ratio of 1:3 by mixing 2.5 mM of the protein solution with 7.5 mM of ferri-desferal and incubated for 30 minutes in ice. Holo-*Vc*FhuD crystals grew similarly with the precipitant solution consisting of 0.8 M ammonium sulfate, 0.1 M HEPES (pH 7.0) after incubation for 20 days against 1.6 M ammonium sulfate, 0.1 M NaCl and 0.1 M Tris (pH 7.0).Crystals were then soaked in cryoprotectant containing 20% (v/v) glycerol, and mother liquor and flash frozen. Crystallization of apo-*Vc*HutB was done as per protocol reported previously^25^.

Diffraction data of apo and holo *Vc*FhuD were collected at 100 K at the PX-BL21^32^ beamline of Indus-2 synchrotron, India with resolutions up to 2.4 Å and 3.4 Å respectively. The data were indexed and integrated using XDS^33^ and subsequently scaled using AIMLESS from CCP4^34^. Data-collection and processing parameters are given in Table 1.

### Phasing and Model Refinement

The initial phases for the PBPs were obtained by molecular replacement (MR) using PHASER^35^. Since, during phasing experiments of apo-*Vc*FhuD no apo structure was available for any such PBP, we used the coordinates of the Gallichrome bound structure of *Ec*FhuD (PDB code: 1EFD) for MR calculations. Two molecules of apo-*Vc*FhuD were obtained in the asymmetric unit (with RFZ = 4.4, TFZ = 11.4 and LLG = 144), in the space group *P*2_1_2_1_2_1_ using data between 47 Å and 2.5 Å resolutions. Few cycles of refinement by PHENIX^36^, systematic model building using *WinCoot*^37^, gradual inclusion of the solvents individual B-factor and TLS refinement produced a final R_cryst_ of 18.9% (R_free_ = 23.7%) (Table 1). Non-crystallographic symmetry (NCS) was not used during the refinement of apo-*Vc*FhuD structure.

Ferri-desferal bound *Vc*FhuD was crystallized in the hexagonal space group P*3*_*2*_*21* (Table 1). MR with the apo-*Vc*FhuD model identified four molecules of ferri-Desferal bound *Vc*FhuD in the asymmetric unit. Four folds of NCS were used during refinement.After few cycles of positional refinement and simulated annealing, ferri-desferal was fitted (using the Desferal coordinates of PDB code 1K2V) in the positive density observed in the 2F_o_-F_c_ map at the ligand binding cleft of all four polypeptide chains. The structure of holo-*Vc*FhuD was refined well with final R_cryst_ of 19.2% (R_free_ = 22.8%) (Table 1).

Coordinates of apo-*Sd*ShuT (PDB code: 2RG7; 20) produced acceptable MR solution for *Vc*HutB. The search model was modified with removal of water molecules, truncation of certain loops around the ligand binding pocket and truncation of the non-identical amino acids to Alanine. One molecule of this modified search model produced the rotation function Z-score (RFZ) of 4.2, translation function Z-score (TFZ) of 10.0 and a convincing log-likelihood gain (LLG) of 88.0 in the space group P*4*_*3*_*2*_*1*_*2* using data between 37.0 Å and 2.4 Å resolutions. Refinement was done in a process similar to apo-*Vc*FhuD with gradual inclusion of the missing regions. Final refinement produced R_work_ and R_free_ of 19.7% and 25.5% respectively (Table 1).

### Fluorescence quenching studies

Fluorescence measurement was carried out using a spectrofluorometer, Hitachi F-7000 following the protocol described in Raines *et al*.^38^. Changes in fluorescence of tryptophan and tyrosine residues were measured at an excitation wavelength of 280 nm and the emission spectra were recorded between 295 nm and 410 nm with slit widths of 5 nm for both excitation and emission. All reactions were carried out at 298 K. The reactions were performed in a buffer containing 50 mM Tris-HCl (pH 7.0) and 300 mM NaCl. Equilibrium titration of *Vc*FhuD was carried out individually with ferrichrome, ferri-desferal and ferri-enterobactin. Desferal, ferrichrome (iron-free) and enterobactin were purchased from Sigma-Aldrich. Purity was of ≥92.5% (TLC grade), ≥98% (TLC grade) and ≥98% (HPLC grade) for desferal, ferrichrome and enterobactin respectively. 15 mM stock solutions of desferal and ferrichrome were prepared in buffer with composition mentioned above while a 15 mM stock solution of enterobactin was prepared in 90% (v/v) acetonitrile. These ligands were treated with FeCl_3_ in 1:2 molar ratio to get Fe^3+^ bound ligands. Ferrichrome, ferri-desferal were then diluted to 300 μM in the same buffer while ferri-enterobactin was diluted to 500 μM in 90% (v/v) acetonitrile to use as stock solutions during experiments.

The concentration of *Vc*FhuD was 1 μM and ligand concentrations varied from 0 to 23 μM. The dissociation constant, *K*_*d*_ was determined using nonlinear curve fitting analysis as per equations [1] and [2]^39^. All experimental points for the binding isotherms were fitted by the least-squares method:


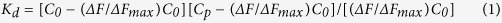



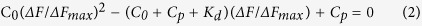


*C*_*0*_ and *C*_*p*_ denotes the input concentrations of the ligands and *Vc*FhuD respectively. *ΔF* is the change in fluorescence intensity at 337 nm (λ_ex_ = 280 nm) for each point of titration curve and *ΔF*_*max*_ is the same parameter when ligand is totally bound to the protein. A double-reciprocal plot of 1/*ΔF* against 1/(*C*_*p*_ *−* *C*_*0*_), as shown in equation [3] was used to determine the *ΔF*_*max*_.


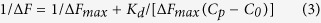


Δ*F*_*max*_ was calculated from the slope of the best-fit line corresponding to the above plot. All experimental data points of the binding isotherm were fitted by linear fit analysis using Microsoft EXCEL and Origin 8.0.

### Circular Dichroism

Circular Dichroism spectroscopy was carried out using a MOS-450 spectrometer, Bio-Kine. The instrument was operated in the near-UV range to study the ferri-enterobactin binding to *Vc*FhuD. The instrument was operated with the following parameters: wavelength range, 250–500 nm; scanning mode, continuous; scanning speed, 100 nm/min; response, 0.5 s; accumulation, 3; pathlength, 1 mm. The experiment was carried out at 298 K. The reactions were performed in a buffer containing 50 mM Tris-HCl (pH 7.0) and 300 mM NaCl. The concentration of *Vc*FhuD was 33 μM and ligand concentration varied from 20 to 300 μM. The blank spectrum was subtracted from all spectra.

### Spectroscopic titration assays

For hemin titrations, 10 μM purified 6 × His-tagged *Vc*HutB in 300 mM NaCl and 50 mM Tris–HCl (pH 7.0 and 8.0) and its mutants *Vc*HutB-Y65A and Y65F in pH 8.0 were subjected to UV-Visible spectroscopy. A 3 mM hemin stock solution was prepared in 100% DMSO. Stock concentration was determined spectrophotometrically (ε385 = 58,400 M^−1^ cm^−1^). Hemin concentration was successively added from 2 μM to 40 μM, into 10 μM of protein. Samples were equilibrated for 10 min after addition of each hemin aliquot and UV/Vis spectra between 250 nm and 700 nm were recorded with a dual-beam spectrophotometer Hitachi U2900 at 293 K. The resulting difference spectra were generated by subtracting the free heme spectra from the heme-titrated *Vc*HutB spectra. Reproducibility of the results was confirmed by performing the experiments in triplicate. The mean values are reported here with errors representing the standard error of the mean at less than 0.05%.

### Native PAGE heme-binding gel shift assay

A 3 mM hemin stock solution was prepared as described above. Purified 6 × His-tagged *Vc*HutB (0.2 mM) in buffer containing 300 mM NaCl, 50 mM Tris (pH 8.0) was incubated with hemin of gradually increasing concentration from 0.025 mM to 1.2 mM or with buffer alone for 45 min at 293 K and subjected to native PAGE. The *Vc*HutB mutants Y65A and Y65F (0.2 mM) in the same buffer were individually incubated with 0 mM, 0.1 mM, 0.2 mM and 0.4 mM hemin for 45 min at 293 K and subjected to native PAGE. Similar incubation experiment was carried out with 6 × His-tagged *Vc*HutB at pH 7.0. Continuous native PAGE was performed as reported previously^27^ and all the experiments were done at least in triplicate. The hemin-complexed protein species migrated faster compared to the apo-forms as was already described for other hemin binding proteins^28^. Therefore, the disappearance of apo-*Vc*HutB as a function of hemin concentration was quantified densitometrically, after Coomassie staining, using Quantity One Software. The fold-densitometric decrease of apo-HutB-band intensity was plotted against hemin concentration and K_d_ was calculated using GraphPad Prism software (www.graphpad.com).

### Molecular dynamics simulation

We carried out MD simulations for 1000 ns on *Vc*HutB and *Vc*FhuD in explicit solvent using Gromacs-5.0.4 program^40^ with CHARMM27^41^ all atom force field. The simulation box was a dodecahedron with minimum protein-edge distance of 12 Å and periodic boundary condition was applied on all the three (xyz) directions. The protein was solvated with TIP3P waters and the system was neutralized by adding suitable numbers of ions (5 Na^+^ for *Vc*HutB and 4 Na^+^ for *Vc*FhuD) replacing the solvent molecules at random locations. After the energy minimization of the whole system using the steepest descent algorithm, the system was gradually heated to 300 K using NVT ensemble. The system was then equilibrated using NPT ensemble. During the equilibrations, the protein backbone was restraint with a harmonic potential of force constant 1000 kJ/mol. The leap-frog integrator with a time-step of 2 fs was used. The Parrinello-Rahman algorithm^42^ was employed to control the pressure at 1 bar with a coupling constant of 2 ps and the modified Berendsen (V-rescale)^43^ thermostat was used to control the temperature of the system at 300 K with a time constant of 0.1 ps. The Particle Mesh Ewald (PME)^44^ method was used to compute the electrostatic interactions with a real space cut-off distance of 12 Å. The same cut-off value was used for calculations of the van der Waals interactions. After 5 ns of equilibration using position restraints on the protein, the production MD simulation run was carried out for 1000 ns. The position and the velocity of all the atoms were recorded in the trajectory file at every 20 ps for analysis of the dynamics. To avoid terminal motion excessively dominate the results, the first and last five residues were not considered for the analysis.

### Equipment and settings

Sequence and structural alignments were carried out using the programs Clustal Omega^45^ and *PyMOL*^46^ respectively. Structure figures were prepared using *PyMOL*^46^. **C**oulombic potential of surfaces were calculated using the program UCSF-chimera^47^. Figures 3 and 5 were prepared using Microsoft EXCEL and Origin 8.0. Native Gels were scanned using MEGA-CAPT software of Gel Documentation System. All the figures were panelled using Adobe Photoshop CS3.

## Additional Information

**Accession Codes**: The atomic coordinates and structure factors of apo-VcFhuD, holo-VcFhuD and apo-VcHutB have been deposited in the Protein Data Bank (http://wwpdb.org/) with PDB codes 5GGY, 5GGX, 5KHL respectively.

**How to cite this article**: Agarwal, S. *et al*. Structure and dynamics of Type III periplasmic proteins *Vc*FhuD and *Vc*HutB reveal molecular basis of their distinctive ligand binding properties. *Sci. Rep.* **7**, 42812; doi: 10.1038/srep42812 (2017).

**Publisher's note:** Springer Nature remains neutral with regard to jurisdictional claims in published maps and institutional affiliations.

## Supplementary Material

Supplementary Figures

## Figures and Tables

**Table 1:** Data collection statistics and refinement statistics.

	Apo-*Vc*FhuD	Desferoxamine mesylate bound *Vc*FhuD	Apo-*Vc*HutB
***Data collection statistics***			
Beamline	PXBL21	PXBL21	PXBL21
Wavelength (Å)	0.980	0.980	0.980
Detector	MarCCD-225	MarCCD-225	MarCCD-225
Oscillation (°)	1	1	1
Space group	*P2*_*1*_*2*_*1*_*2*_*1*_	*P3*_*2*_*21*	*P4*_*3*_*2*_*1*_*2*
Unit-cell parameters (Å)	a = 57.25, b = 71.75, c = 125.89	a = b = 191.15, c = 129.16	a = b = 62.88, c = 135.8
Resolution (Å)	47.32–2.50 (2.60–2.50)	47.79–3.40 (3.55–3.40)	37.2–2.4 (2.49–2.40)
No. of molecules per asymmetric unit	2	4	1
R_pim_ (%)	9.2 (41.6)	7.1 (34.1)	2.1 (24.6)
Average I/σ(I)	9.1(2.2)	10.0 (2.1)	29.9 (3.3)
Completeness (%)	100 (100)	100 (100)	99.2 (97.5)
Multiplicity	7.3 (7.4)	11.2 (11.4)	9.7 (9.5)
***Refinement statistics***			
Resolution (Ǻ)	2.5–47.316	3.4–43.055	2.39–28.53
Number of reflections	18616	37750	11157
R_work_/R_free_ (%)	18.9/23.7	19.2/22.8	19.7/25.5
No. of atoms			
All	4324	8439	1968
Proteins	4152	8299	1915
Ligand	2	140	5
Water	170	0	48
B-factor (Å^2^)			
Average			
Protein	33.2	77.4	62.7
Ligand	38.1	94.0	71.1
Water	32.2	0	59.5
RMSDs Bond lengths/bond angles (Å/°)	0.003/0.729	0.003/0.861	0.009/1.298
Ramachandran statistics (%)			
Most favoured	96.41	92.88	94.92
Additionally allowed	3.40	5.41	4.30
Disallowed	0.19	1.71	0.78

*Values in parentheses are for the outer-shell.*
