# Supplementary material for: Structure and dynamics of Type III periplasmic proteins VcFhuD and VcHutB reveal molecular basis of their distinctive ligand binding properties
Source: Sci Rep. 2017 Feb 20;7:42812. doi: 10.1038/srep42812 (PMC5316997; doi:10.1038/srep42812)
Supplement: Supplementary Figures [file srep42812-s1.doc]

**Structure and dynamics of Type III periplasmic proteins *Vc*FhuD and *Vc*HutB reveal molecular basis of their distinctive ligand binding properties**

***Shubhangi Agarwal^1^, Sanjay Dey^1^, Biplab Ghosh^2^, Maitree Biswas^1,3^ and Jhimli Dasgupta^1,*^***

^1^Department of Biotechnology, St. Xavier’s College, 30 Park Street, Kolkata 700016, India

^2^High Pressure & Synchrotron Radiation Physics Division, Bhabha Atomic Research Centre, Trombay, Mumbai 400085, India

^3^Current address: University of British Columbia, Dept of Biochemistry and Molecular Biology, LSI, Rm #5340, 2350 Health Sciences Mall, Vancouver, BC, Canada

*To whom correspondence should be addressed: Dept. of Biotechnology, St. Xavier’s College, 30 Park St., Kolkata 700016, India. Tel.: 91-33-22551275; Fax: 91-33-22879966; E-mail: [jhimli@sxccal.edu](mailto:jhimli@sxccal.edu)


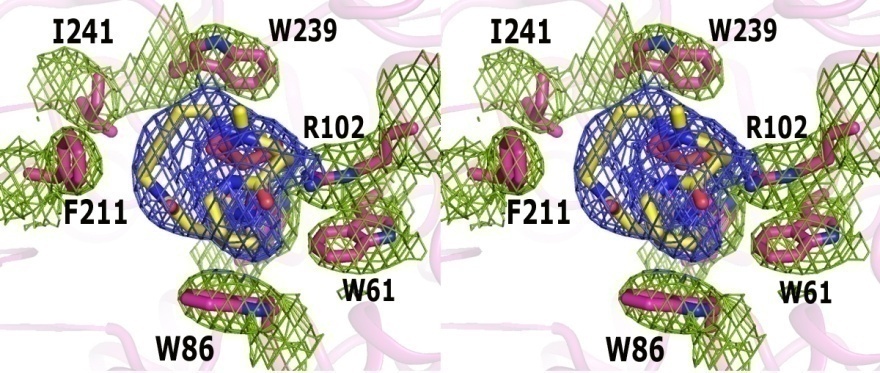


**Figure S1:** Stereo view of 2*Fo*−*Fc* electron density map (contoured at 1σ) around the bound ferri-desferal and the interacting residues of *Vc*FhuD.


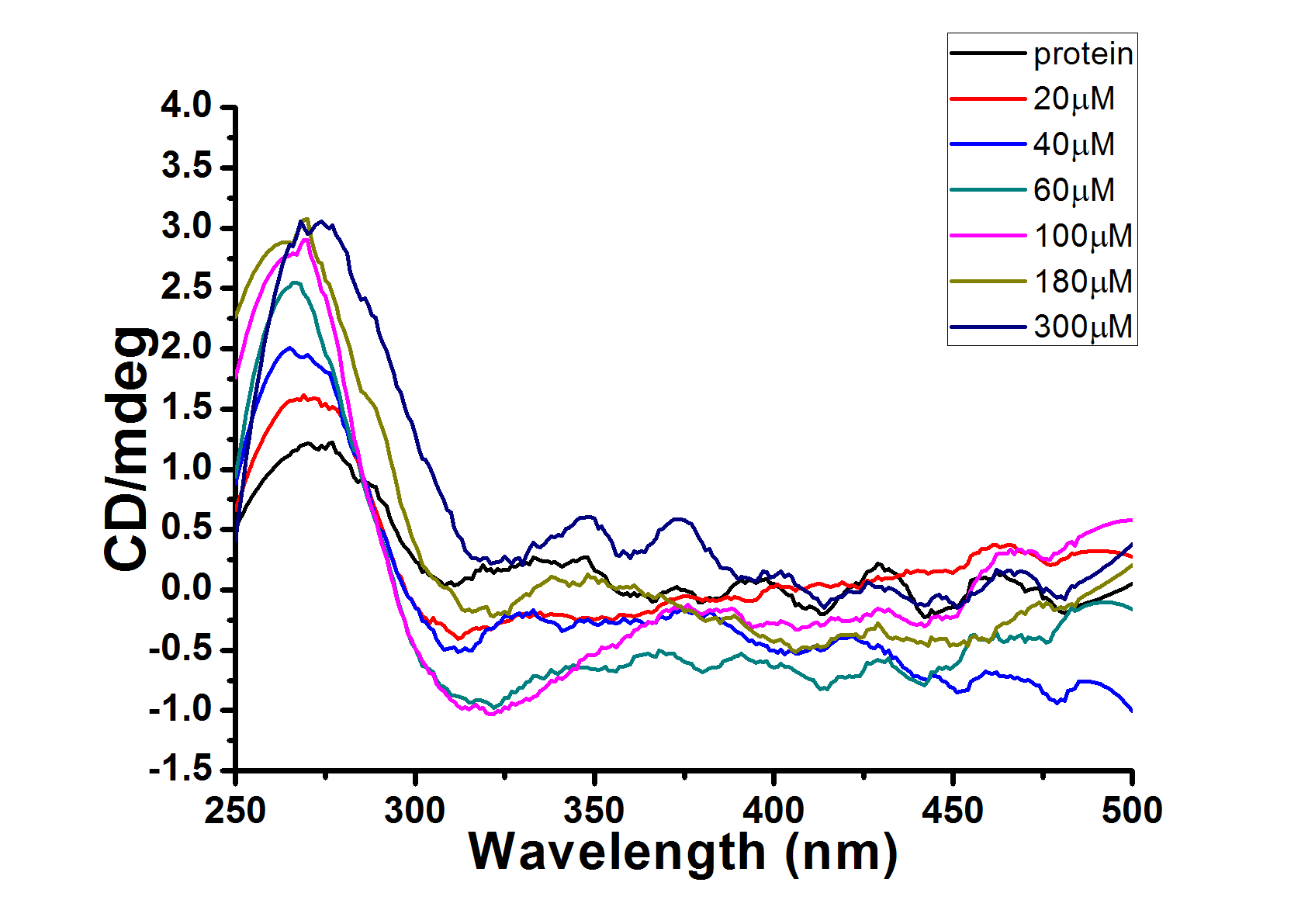


**Figure S2:** CD spectra recorded on addition of increasing amount of Ferri-enterobactin to *Vc*FhuD.


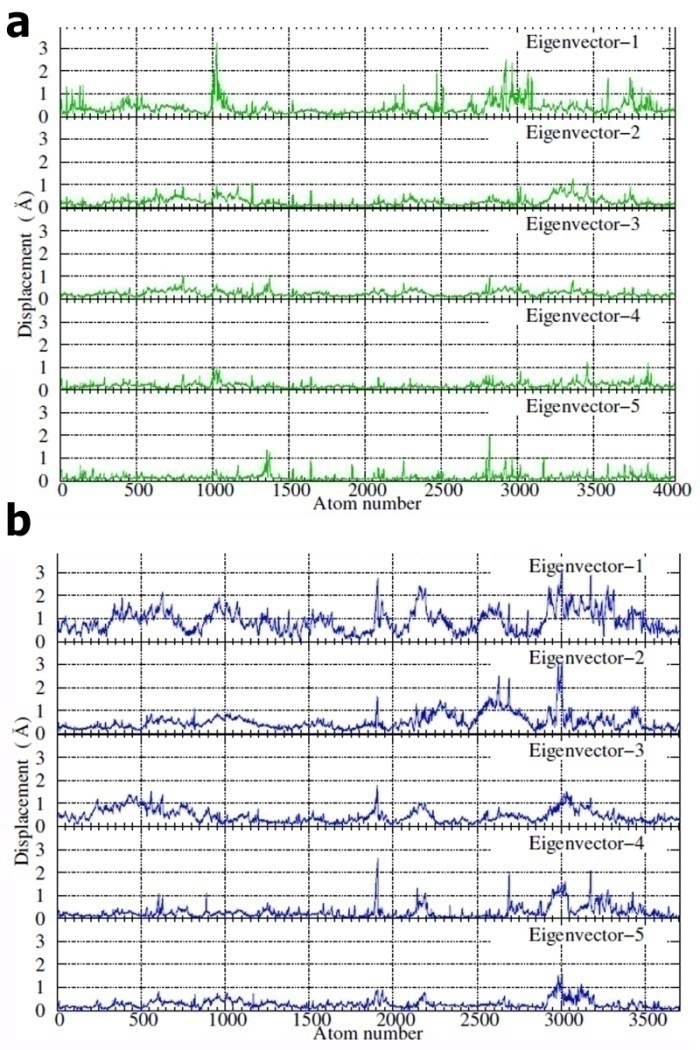


**Figure S3:** The motions along the first five eigenvector directions for (a) *Vc*FhuD (b) *Vc*HutB are shown by projecting the trajectory against time.
